# Association between anesthesia duration and outcome in dogs with surgically treated acute severe spinal cord injury caused by thoracolumbar intervertebral disk herniation

**DOI:** 10.1111/jvim.15796

**Published:** 2020-05-17

**Authors:** Joe Fenn, Hongyu Ru, Nick D. Jeffery, Sarah Moore, Andrea Tipold, Franz J. Soebbeler, Adriano Wang‐Leandro, Christopher L. Mariani, Peter J. Early, Karen R. Muñana, Natasha J. Olby

**Affiliations:** ^1^ Department of Clinical Science and Services Royal Veterinary College Hertfordshire United Kingdom; ^2^ The Canine Spinal Cord Injury Consortium (CANSORT‐SCI); ^3^ Department of Statistics North Carolina State University Raleigh North Carolina USA; ^4^ Department of Small Animal Clinical Sciences College of Veterinary Medicine and Biomedical Sciences, Texas A&M University College Station Texas USA; ^5^ Department of Veterinary Clinical Sciences The Ohio State University College of Veterinary Medicine Columbus Ohio USA; ^6^ Department of Small Animal Medicine and Surgery University of Veterinary Medicine Hannover Germany; ^7^ Department of Clinical Sciences College of Veterinary Medicine, North Carolina State University Raleigh North Carolina USA

**Keywords:** canine, extrusion, hemilaminectomy, prognosis, surgery

## Abstract

**Background:**

Retrospective research recently identified a possible relationship between duration of surgery and outcome in severely affected dogs treated surgically for acute thoracolumbar intervertebral disk herniation (TL‐IVDH).

**Hypothesis:**

That increased duration of surgery is associated with poorer outcome in dogs with absent pain perception treated surgically for TL‐IVDH.

**Animals:**

Two hundred ninety‐seven paraplegic dogs with absent pain perception surgically treated for acute TL‐IVDH.

**Methods:**

Retrospective cohort study. Medical records of 5 institutions were reviewed. Inclusion criteria were paraplegia with absence of pain perception, surgical treatment of TL‐IVDH, and 1‐year postoperative outcome (ambulatory: yes or no). Canine data, outcome, and surgery and total anesthesia duration were retrieved.

**Results:**

In this study, 183/297 (61.6%) dogs were ambulatory within 1 year, 114 (38.4%) dogs failed to recover, including 74 dogs (24.9%) euthanized because of progressive myelomalacia. Median anesthesia duration in dogs that regained ambulation within 1 year of surgery (4.0 hours, interquartile range [IQR] 3.2‐5.1) was significantly shorter than those that did not (4.5 hours, IQR 3.7‐5.6, *P* = .01). Multivariable logistic regression demonstrated a significant negative association between both duration of surgery and total anesthesia time and ambulation at 1 year when controlling for body weight and number of disk spaces operated on.

**Conclusions and Clinical Importance:**

Findings support a negative association between increased duration of anesthesia and outcome in this group of dogs. However, the retrospective nature of the data does not imply a causal relationship.

**Abbreviations**
CT: computed tomography
IVDH: intervertebral disk herniation
MRI: magnetic resonance imaging
NCSU: North Carolina State University
OSU: Ohio State University
PMM: progressive myelomalacia
RVC: Royal Veterinary College
SCI: spinal cord injury
TAMU: Texas A&M University
UVMH: University of Veterinary Medicine Hannover

## 1 INTRODUCTION

Acute thoracolumbar intervertebral disk herniation (IVDH) is the leading cause of acute spinal cord injury (SCI) in dogs.^1^ Spinal cord injury after acute IVDH occurs through a combination of contusive and compressive insults, leading to a cascade of progressive secondary cellular and biochemical injury pathways.^1^, ^2^, ^3^ Treatment can be either medical or surgical, with the decision typically based on the severity of presenting clinical signs.^4^, ^5^ The most severely affected dogs are those with paraplegia and absent pain perception. Dogs presenting with this degree of severity typically undergo surgical treatment by hemilaminectomy in order to decompress the affected spinal cord.^5^, ^6^ Successful outcome rates for surgical treatment of dogs with absent pain perception secondary to thoracolumbar IVDH range from 25% to 76%, compared to close to 100% for less severely affected dogs.^4^, ^6^, ^7^, ^8^, ^9^ Whereas the variability of recovery within the group of dogs with absent pain perception can be ascribed in part to differences in initial injury severity captured by measurement of serum concentrations of glial fibrillary acidic protein and phosphorylated neurofilament heavy chain at time of injury,^10^, ^11^, ^12^ as well as extent of injury seen on magnetic resonance imaging (MRI),^7^ it is important to examine factors associated with management of these cases that could influence their ultimate outcome.

Of the many factors that contribute to the secondary injury cascade initiated by SCI, spinal cord perfusion appears to be one of the most important variables.^13^, ^14^ Indeed, outcome after experimental acute SCI correlates with the perfusion of the injured spinal cord.^2^, ^15^, ^16^ As spinal cord perfusion in SCI is heavily dependent on systemic blood pressure, perfusion variables remain a vital consideration in treatment of acute SCI both in veterinary and human medicine as well as under experimental conditions.^3^, ^14^, ^17^, ^18^, ^19^, ^20^, ^21^ Given the established hemodynamic consequences of general anesthesia in dogs, including a high prevalence of bradycardia and hypotension,^22^, ^23^ exposure to these changes could have a negative impact on outcome. Although there have been variable results regarding the effect of these specific intraoperative variables on outcome after IVDH reported in the veterinary literature,^24^, ^25^ increasing total duration of anesthesia and surgery might be negatively associated with outcome in the most severely affected dogs, those with absent pain perception.^9^ The recently established Canine Spinal Cord Injury Consortium (CANSORT‐SCI) was created with the aim of facilitating large‐scale research into acute SCI in dogs, through collaboration between multiple international institutions.^26^, ^27^ This initiative has provided an opportunity to investigate hypotheses generated by exploratory research such as this in a large, diverse population of dogs across different institutions and countries.

The aim of this study was to investigate whether there was an association between outcome and anesthesia duration using the CANSORT‐SCI to provide access to a large population of severely affected dogs surgically treated for thoracolumbar IVDH at multiple international institutions.^26^ Our hypothesis was that there would be a negative association between duration of anesthesia and outcome in dogs with absent pain perception treated surgically for thoracolumbar IVDH.

## 2 MATERIALS AND METHODS

### 2.1 Study design and animals

This retrospective cohort study included cases from 5 institutions (North Carolina State University [NCSU], Royal Veterinary College [RVC], Ohio State University [OSU], Texas A&M University [TAMU], University of Veterinary Medicine Hannover [UVMH]) recruited through the international CANSORT‐SCI network.^26^ Inclusion criteria were dogs that had undergone surgical treatment by hemilaminectomy for thoracolumbar IVDH; paraplegic with absent pain perception in both pelvic limbs and tail before surgery; available anesthesia duration records (total general anesthesia duration and surgery duration); with 1‐year ambulatory status follow‐up recorded. Exclusion criteria were incomplete anesthesia duration records or lack of follow‐up data, with dogs euthanized owing to signs of progressive myelomalacia (PMM) postoperatively included as an unsuccessful (nonambulatory) outcome.

Case records were retrospectively analyzed for descriptive dog (breed, age, body weight, and sex) and clinical data (imaging modality used, surgery site, and number of intervertebral disk [IVD] spaces operated on). Duration of surgery (first incision to last suture) and total duration of anesthesia (administration of induction agent to extubation) were also retrieved from the medical records. Outcome was determined according to ambulatory or nonambulatory status 1 year postoperatively, with ambulatory function defined as the ability to take at least 10 consecutive fully weight‐bearing steps without falling over on reexamination. Dogs euthanized postoperatively because of the development of clinical signs suggestive of PMM were included in the nonambulatory outcome category.

Continuous variables were analyzed for normality using Shapiro‐Wilk tests. Descriptive data were reported as mean (±SD) for normally distributed variables and median (minimum, maximum, interquartile range [IQR]) for non‐normally distributed data. Mann‐Whitney *U* tests were used to compare duration of anesthesia and surgery in dogs that regained ambulatory status and those that did not. Based on the possible influence of the number of IVD spaces included in the hemilaminectomy (extent of surgery) and body weight on duration of anesthesia, the effect of these 2 variables on anesthesia and surgery time were evaluated using Pearson correlation coefficients. These variables were then included alongside surgery and anesthesia duration in 2 separate multiple logistic regression models to predict recovery of ambulation 1 year postoperatively, with goodness of fit evaluated using Hosmer and Lemeshow tests. To evaluate these findings in a population with confirmed 1‐year follow‐up in all dogs, this analysis was also repeated with the exclusion of cases euthanized owing to signs of PMM in the postoperative period. Statistical significance was established as *P* < .05 where relevant. All data analysis was performed using commercially available statistical software programs (SPSS v22, IBM SPSS Inc, Chicago, Illinois; SAS, SAS Institute, Cary, North Carolina; GraphPad Prism Version 7.0d for Mac OS X, GraphPad Software, La Jolla, California).

## 3 RESULTS

### 3.1 Population

Data were available for 297 dogs that fulfilled the inclusion criteria, from NCSU (n = 162, including 35 dogs used in a previous retrospective study^9^), TAMU (n = 62), OSU (n = 36), RVC (n = 29), and UVMH (n = 8) between 2004 and 2017. There were 35 different breeds included, with Dachshund being the most common breed (n = 161, 54.2%), followed by mixed breeds (n = 36, 12.1%). A full list of breeds is included in Supporting Information. One hundred fifty‐seven dogs (52.9%) were male (105 neutered) and 140 (47.1%) were female (114 neutered). The median age was 5.0 years (minimum 2.0, maximum 15.0, IQR 4.0‐6.0) and median body weight was 7.5 kg (minimum 2.4, maximum 52, IQR 5.7‐10.4).

One hundred twenty‐one dogs (40.7%) underwent MRI, 118 (39.7%) computed tomography (CT), 38 (12.8%) myelography, and 14 (4.7%) CT‐myelography. Four dogs (1.4%) underwent both MRI and CT and for 2 dogs (0.7%), the imaging modality was not available. A single site hemilaminectomy was performed in 180 dogs (60.6%), with 85 dogs (28.6%) undergoing a 2‐space hemilaminectomy and 32 dogs (10.8%) undergoing a hemilaminectomy over more than 2 intervertebral spaces. Median total anesthesia duration across all dogs was 4.2 hours (minimum 1.5, maximum 10.9, IQR 3.3‐5.3), with a median surgery duration of 2.2 hours (minimum 0.7, maximum 7.8, IQR 1.5‐3.0).

### 3.2 Outcome

One hundred eighty‐three (61.6%) of 297 dogs were ambulatory within 1 year postoperatively, while 114 dogs (38.4%) failed to regain ambulatory function within 1 year. Seventy‐four dogs (24.9%) were euthanized postoperatively because of the development of clinical signs suggestive of PMM, with these dogs included in the nonambulatory category for comparison between outcome groups.

**FIGURE 1:**
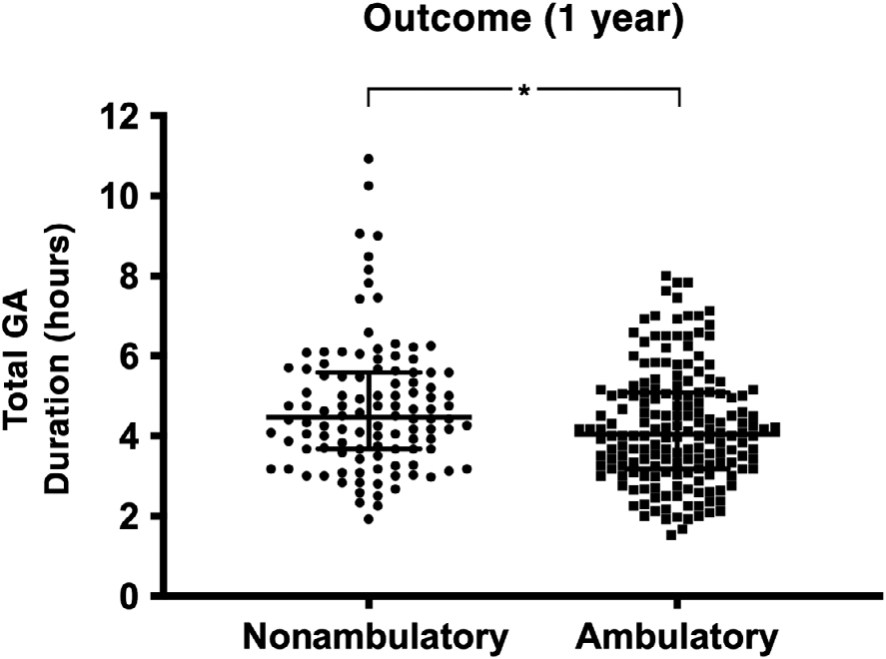
Dot plots showing the distribution of total anesthesia duration (Total general anesthesia (GA) duration) for 297 dogs with absent pain perception that were ambulatory (n = 183) or nonambulatory (n = 114) within 1 year of underdoing surgical treatment for acute thoracolumbar intervertebral disk herniation. Central line = median, whiskers = interquartile range. *Statistically significant difference (*P* = .010)

**FIGURE 2:**
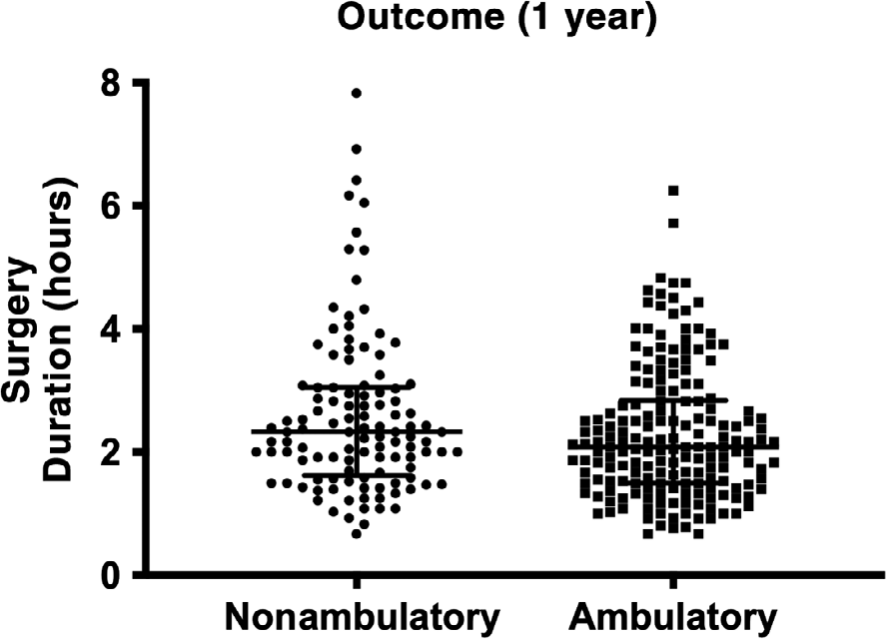
Dot plots showing the distribution of surgery duration for 297 dogs with absent pain perception that were ambulatory (n = 183) or nonambulatory (n = 114) within 1 year of underdoing surgical treatment for acute thoracolumbar intervertebral disk herniation. Central line = median, whiskers = interquartile range. The difference in surgery duration between outcome groups was not statistically significant (*P* = .057)

In dogs that regained ambulatory function within 1 year postoperatively, total duration of anesthesia was significantly shorter, with a median of 4.0 hours (minimum 1.5, maximum 8.0, IQR 3.2‐5.1), compared to 4.5 hours (minimum 1.9, maximum 10.9, IQR 3.7‐5.6) in those that did not (*P* = .01; Figure 1). Median duration of surgery in dogs that regained ambulatory function within 1 year was 2.1 hours (minimum 0.7, maximum 6.3, IQR 1.5‐2.8), while in dogs that did not recover ambulation it was 2.3 hours (minimum 0.7, maximum 7.8, IQR 1.6‐3.1). The duration of surgery was not significantly different between the 2 groups on bivariate analysis (*P* = .06; Figure 2). The median total duration of anesthesia for dogs that were euthanized postoperatively for presumed PMM was 4.3 hours (minimum 1.9, maximum 8.5, IQR 3.7‐5.5) and the median duration of surgery was 2.3 hours (minimum 1.0, maximum 6.4, IQR 1.5‐2.0). When repeating the analysis after excluding cases euthanized for PMM, duration of anesthesia remained significantly shorter (median 4.0 hours, minimum 1.5, maximum 8.0, IQR 3.2‐5.1) in 183 dogs that recovered ambulatory function compared to in the 40 dogs that did not (median 4.8 hours, minimum 2.3, maximum 10.9, IQR 3.8‐5.9; *P* < .001).

The scatterplots in Figures 1 and 2 demonstrate several marked outliers in the nonambulatory outcome group. Six dogs underwent an anesthesia for longer than 8 hours, with the surgery time ranging from 5.3 to 7.8 hours (median 6.1 hours), all of which had an unsuccessful outcome. Eleven dogs overall had a surgical duration of more than 5 hours, with just 2 of these regaining ambulatory function within 1 year and 2 developing PMM. All of these dogs except 1, underwent multilevel hemilaminectomies over a median of 3 IVD spaces (ranging from 1 to 5 spaces). They were also larger dogs overall, with a median body weight of 17.5 kg (minimum 3.4, maximum 38, IQR 7.0‐26.2) compared to the overall study population median of 7.5 kg (minimum 2.4, maximum 52, IQR 5.7‐10.4).

There was a significant positive correlation between body weight and both anesthesia (*P* < .001, *r* = 0.435) and surgery duration (*P* < .001, *r* = .367), as well as between number of IVD spaces operated and anesthesia (*P* < .001, *r* = .365) and surgery duration (*P* < .001, *r* = .394). Based on these results, these variables were included as covariates in multiple regression models to predict ambulatory status postoperatively as shown in Tables 1 and 2. The models demonstrate that, when controlling for body weight and number of intervertebral disk spaces operated, for each 1 hour increase in surgery (odds ratio [OR] 0.751, *P* = .02) and total anesthesia duration (OR 0.751, *P* = .003) there was a 24.9% and 27.2% decrease in the odds of regaining ambulatory status within 1 year, respectively. Tables 3 and 4 show the same models, with similar results, performed after excluding cases that were euthanized owing to signs of PMM.

**TABLE 1:** Multiple logistic regression model including anesthesia duration, body weight, and size of hemilaminectomy as predictors of ambulatory status 1 year postoperatively among 297 dogs with absent pain perception that underwent surgical treatment for acute thoracolumbar intervertebral disk herniation

Variable	Odds ratio	95% CI		*P* value
Duration of anesthesia (h)	0.751	0.632	0.906	.003*
Body weight (kg)	1.037	0.997	1.078	.071
Number of IVD spaces operated	0.918	0.662	1.273	.61

*Note:* Hosmer and Lemeshow test χ^2^ = 13.4, *P* = .10.

Abbreviations: CI, confidence interval; IVD, intervertebral disk.

* Statistically significant (*P* < .05).

**TABLE 2:** Multiple logistic regression model, including surgery duration, body weight, and size of hemilaminectomy as predictors of ambulatory status 1 year postoperatively among 297 dogs with absent pain perception that underwent surgical treatment for acute thoracolumbar intervertebral disk herniation

Variable	Odds ratio	95% CI		*P* value
Duration of surgery (h)	0.751	0.596	0.946	.015*
Body weight (kg)	1.031	0.993	1.071	.11
Number of IVD spaces operated	0.925	0.671	1.277	.64

*Note:* Hosmer and Lemeshow test χ^2^ = 2.9, *P* = .94.

Abbreviations: CI, confidence interval; IVD, intervertebral disk.

* Statistically significant (*P* < .05).

**TABLE 3:** Multiple logistic regression model including anesthesia duration, body weight, and size of hemilaminectomy as predictors of ambulatory status 1 year postoperatively among 223 dogs with absent pain perception that underwent surgical treatment for acute thoracolumbar intervertebral disk herniation—excluding cases euthanized for presumed progressive myelomalacia

Variable	Odds ratio	95% CI		*P* value
Duration of anesthesia (h)	0.675	0.521	0.875	.003*
Body weight (kg)	1.030	0.977	1.086	.27
Number of IVD spaces operated	0.876	0.571	1.345	.55

*Note:* Hosmer and Lemeshow test χ^2^ = 9.4, *P* = .31.

Abbreviations: CI, confidence interval; IVD, intervertebral disk.

* Statistically significant (*P* < .05).

**TABLE 4:** Multiple logistic regression model including surgery duration, body weight, and size of hemilaminectomy as predictors of ambulatory status 1 year postoperatively among 223 dogs with absent pain perception that underwent surgical treatment for acute thoracolumbar intervertebral disk herniation—excluding cases euthanized for presumed progressive myelomalacia

Variable	Odds ratio	95% CI		*P* value
Duration of surgery (h)	0.723	0.531	0.984	.039*
Body weight (kg)	1.015	0.966	1.067	.56
Number of IVD spaces operated on	0.870	0.572	1.324	.52

*Note:* Hosmer and Lemeshow test χ^2^ = 1.9 *P* = .98.

Abbreviations: CI, confidence interval; IVD, intervertebral disk.

* Statistically significant (*P* < .05).

## 4 DISCUSSION

The findings of this study provide further evidence of a negative association between increased duration of anesthesia and recovery of ambulation in severely affected dogs surgically treated for thoracolumbar IVDH. We were able to explore and further investigate the findings of recent retrospective research using data from a large cohort of dogs with absent pain perception recruited through the international CANSORT‐SCI network of referral hospitals.^26^, ^27^ Although this association could have important implications in this cohort of the most severely affected dogs undergoing surgical treatment for thoracolumbar IVDH, the results of this study do not confirm a causal relationship between anesthesia duration and outcome. Several individual cases with extremely prolonged durations of anesthesia and surgery were present within the study cohort and likely affected the results of the statistical analyses. The potential for a causal relationship requires further investigation, with a particular focus on the factors involved in those cases with a markedly increased duration of anesthesia or surgery.

The cohort of dogs included in the current study represents a large number in comparison to similar studies in the veterinary literature. Using data from 5 different international institutions provided a realistic sample representation by capturing institutional variations in anesthesia protocol and surgeon technique. The dog population was consistent with previously reported characteristics for thoracolumbar IVDH, with Dachshunds comprising over half of the dogs, a slight male predisposition and dogs typically young to middle‐aged at diagnosis.^28^ The majority of dogs underwent advanced imaging for diagnosis of IVDH with either MRI or CT, likely reflecting a trend toward imaging modalities with benefits in terms of diagnostic quality and safety, compared to traditional methods such as myelography.^29^, ^30^, ^31^ Although most dogs subsequently underwent a single site hemilaminectomy (59.4%), 28.6% underwent a 2‐space surgery and 10.8% underwent greater than 2 space hemilaminectomies, allowing us to analyze a diverse population in terms of surgical and anesthesia duration.

Outcome was consistent with previous literature, with 62.0% of the dogs regaining ambulatory function within 1 year postoperatively.^4^, ^6^, ^7^, ^8^, ^9^ In this cohort of dogs with absent pain perception, 74 dogs (24.9%) were euthanized because of the development of clinical signs suggestive of PMM postoperatively, which is more than previous reports of around 10%.^4^, ^28^ However, a recent clinical trial across multiple different institutions found a similar incidence of 17.5%,^32^ while there are differences between breeds with up to 33% of French bulldogs with absent pain perception developing PMM.^33^

Overall average duration of anesthesia and surgery revealed a median of 4.2 hours of anesthesia (IQR 3.3‐5.3) and 2.2 hours of surgery (IQR 1.6‐3.0). Although there was a lack of statistical significance on the bivariate comparison of surgery duration between dogs that did and did not recover, total duration of anesthesia was significantly shorter in dogs that regained ambulatory function (as shown in Figure 1 and supported by the multivariable analysis in Tables 1, 2, 3, 4). Given the association between both the size of the hemilaminectomy performed (number of IVD spaces) and body weight and duration of anesthesia, these variables were included in multivariable analyses to control for any effect on outcome. Multiple logistic regression demonstrated that when controlling for these covariates, both total general anesthesia and surgery duration were significantly shorter in dogs that regained ambulatory status postoperatively (Tables 1 and 3). This provides further support for the findings of our previous retrospective study,^9^ although in a much larger cohort of dogs from multiple international institutions. Furthermore, the results of this study suggest that for each additional hour of surgery and total anesthesia duration, there is a 24.9% and 27.2% decrease, respectively, in the odds of regaining ambulatory status within 1 year.

Numerous factors could be playing a role in driving this relationship, both directly related to the anesthesia, such as cardiovascular variables and perfusion status, as well as other factors such as surgical challenges and complications, such as hemorrhage or prolonged spinal cord manipulation. Other potential confounding factors we considered included body weight and number of surgical sites. There is evidence that larger dogs have a worse outcome than smaller dogs and that longitudinal extent of SCI affects outcome,^4^, ^7^, ^34^ so we controlled for both factors by including them in the logistic regression model. It is important to consider that initial injury severity is the most important predictor of outcome,^4^, ^6^, ^35^ and while all dogs included presented with the same functional grade of injury, this grade will include a wide variation of injury severities. Cases of particularly large volume, explosive IVD extrusions could have caused both a more severe SCI as well as a more challenging and therefore prolonged surgery. It could be hypothesized that this could then lead to an association between prolonged surgery (and anesthesia) and poorer outcome without the cardiovascular or hemodynamic effects of anesthesia being the cause. Further investigation using prospective data collection is required to further elucidate the nature of this relationship.

When considering further the effects of anesthesia on the injured spinal cord, recent studies have shown that this is a population of dogs that experience a high frequency of potentially detrimental cardiovascular changes under anesthesia such as bradycardia and hypotension.^9^, ^25^ After SCI spinal cord vascular autoregulation is impaired, causing blood flow to become directly dependent on systemic blood pressure, with the potential therefore for reduced spinal cord perfusion in these dogs.^16^, ^36^ Although as discussed, the association found in this study does not confirm causality, it could suggest that in cases of markedly prolonged anesthesia, increased exposure to suboptimal conditions for spinal cord perfusion might negatively affect recovery in dogs with severe SCI secondary to IVDH. Future prospective studies could use continuous monitoring under standardized anesthesia and surgical protocols to investigate this further.

Our data include several outliers with these dogs experiencing extreme durations of anesthesia and surgery, as shown in Figures 1 and 2. Of the 6 dogs that underwent general anesthesia for longer than 8 hours and 11 dogs that underwent surgery for longer than 5 hours, only 2 regained ambulatory function. From our retrospective data, it is currently unclear whether this is because of the severity of their initial SCI or factors associated with prolonged anesthesia. This finding warrants further, ideally prospective, investigation to explore these particular cases in more detail. We also repeated the statistical analysis of the study population with PMM cases excluded, with the difference in surgery and anesthesia duration between dogs remaining strongly statistically significant. This suggests that the associations found in this study are not driven by the poorly understood pathophysiological mechanisms that lead to the development of PMM in the postoperative period.^37^

The retrospective nature of this study led to some expected limitations including missing data points and exclusion of several cases owing to a lack of outcome information. Because of the nature of the multicenter data collection, long‐term outcome was restricted to ambulatory status without confirmed pain perception status, meaning that some of the dogs in the recovery group could have developed “spinal walking.” Given the evidence that dogs that recover ambulation without pain perception have intact trans‐lesion connections and smaller lesions than dogs that do not recover ambulation, our emphasis was on regaining the ability to ambulate regardless of pain perception status in the long term as the primary outcome measure for success in such cases.^38^, ^39^, ^40^ We were also not able to access detailed anesthesia protocols including specific medications that were used between institutions. However, the use of the CANSORT‐SCI network allowed the collection of data from several institutions, helping to counteract these limitations. As dogs were treated at 5 different institutions, there might have been variations in surgical technique, anesthetic protocols, and management between centers.

In conclusion, the findings of this study suggest that an increased duration of anesthesia is negatively associated with outcome in paraplegic dogs with absent pain perception undergoing surgical treatment for thoracolumbar IVDH. Although further investigation is required to determine if this is a causal relationship, these results suggest that markedly prolonged anesthesia negatively might affect recovery in severely affected dogs with thoracolumbar IVDH.

## 5 CONFLICT OF INTEREST DECLARATION

Authors declare no conflict of interest.

## 6 OFF‐LABEL ANTIMICROBIAL DECLARATION

Authors declare no off‐label use of antimicrobials.

## 7 INSTITUTIONAL ANIMAL CARE AND USE COMMITTEE (IACUC) OR OTHER APPROVAL DECLARATION

Authors declare no IACUC or other approval was needed.

## 8 HUMAN ETHICS APPROVAL DECLARATION

Authors declare human ethics approval was not needed for this study.

## Supporting information


**Appendix** **S1:** Supporting information

**Appendix** **S2:** Supporting information
