# Supplementary material for: Association between anesthesia duration and outcome in dogs with surgically treated acute severe spinal cord injury caused by thoracolumbar intervertebral disk herniation
Source: J Vet Intern Med. 2020 May 17;34(4):1507–13. doi: 10.1111/jvim.15796 (PMC7379036; doi:10.1111/jvim.15796)
Supplement: Supplementary file 1 — Appendix S1: Supporting information [file JVIM-34-1507-s001.pdf]

Members of CANSORT SCI

Sarah Moore; Natasha Olby; Jonathon Levine; Nick Jeffery; Ronaldo DaCosta; Andrea Tipold; Yvette Nout-Lomas; Ingo Spitzbarth; Nicolas Granger; Veronika Stein; Ji-Hey Lim, Melissa Lewis; Joe Fenn; Holger Volk.
