# Supplementary material for: Association between anesthesia duration and outcome in dogs with surgically treated acute severe spinal cord injury caused by thoracolumbar intervertebral disk herniation
Source: J Vet Intern Med. 2020 May 17;34(4):1507–13. doi: 10.1111/jvim.15796 (PMC7379036; doi:10.1111/jvim.15796)
Supplement: Supplementary file 2 — Appendix S2: Supporting information [file JVIM-34-1507-s002.pdf]

Supplementary Information 1 – List of dog breeds included

Breed	n	%
Dachshund	161	54.2
Mixed breed	36	12.1
English Cocker Spaniel	12	4.0
Pekingese	8	2.7
Pembroke Welsh Corgi	6	2.0
French Bulldog	5	1.7
Beagle	6	2.0
Shih Tzu	5	1.7
Jack Russell Terrier	5	1.7
Toy Poodle	4	1.3
Standard Poodle	3	1.0
Basset Hound	4	1.3
Bichon Frise	2	0.7
Labrador Retriever	3	1.0
Chihuahua	4	1.3
Yorkshire Terrier	4	1.3
American Pitt Bull Terrier	3	1.0
Maltese Terrier	2	0.7
American Cocker Spaniel	3	1.0
Rottweiler	3	1.0
Pomeranian	2	0.7
Schnauzer	2	0.7
Boston Terrier	2	0.7
Coton De Tulear	1	0.3
Rhodesian Ridgeback	1	0.3
American Staffordshire Bull Terrier	1	0.3
Patterdale Terrier	1	0.3
Toy Fox Terrier	1	0.3
Labradoodle	1	0.3
Cairn Terrier	1	0.3
Golden Retriever	1	0.3
Affenpinscher	1	0.3
Papillon	1	0.3
Border Collie	1	0.3
Weimeraner	1	0.3
